# Systematic literature review on the application of machine learning for the prediction of properties of different types of concrete

**DOI:** 10.7717/peerj-cs.1853

**Published:** 2024-05-16

**Authors:** Syeda Iqra Hassan, Sidra Abid Syed, Syed Waqad Ali, Hira Zahid, Samia Tariq, Mazliham Mohd Su ud, Muhammad Mansoor Alam

**Affiliations:** 1 Electrical/Electronic Engineering, British Malaysian Institute, Universiti of Kuala Lumpur, Kuala Lumpur, Malaysia; 2 Electrical Engineering, Ziauddin University, Karachi, Sindh, Pakistan; 3 Biomedical Engineering, Sir Syed University of Engineering and Technology, Karachi, Sindh, Pakistan; 4 Biomedical Engineering, Ziauddin University, Karachi, Sindh, Pakistan; 5 Civil Engineering, Ziauddin University, Karachi, Sindh, Pakistan; 6 Faculty of Computing and Informatics, Multimedia University, Cyberjaya, Selangor, Malaysia; 7 Faculty of Computing, Riphah International University, Islamabad, Pakistan

**Keywords:** Concrete, Machine learning, Compressive strength, Neural network, Mechanical properties, Computer vision, Artificial intelligence, Durability

## Abstract

**Background:**

Concrete, a fundamental construction material, stands as a significant consumer of virgin resources, including sand, gravel, crushed stone, and fresh water. It exerts an immense demand, accounting for approximately 1.6 billion metric tons of Portland and modified Portland cement annually. Moreover, addressing extreme conditions with exceptionally nonlinear behavior necessitates a laborious calibration procedure in structural analysis and design methodologies. These methods are also difficult to execute in practice. To reduce time and effort, ML might be a viable option.

**Material and Methods:**

A set of keywords are designed to perform the search PubMed search engine with filters to not search the studies below the year 2015. Furthermore, using PRISMA guidelines, studies were selected and after screening, a total of 42 studies were summarized. The PRISMA guidelines provide a structured framework to ensure transparency, accuracy, and completeness in reporting the methods and results of systematic reviews and meta-analyses. The ability to methodically and accurately connect disparate parts of the literature is often lacking in review research. Some of the trickiest parts of original research include knowledge mapping, co-citation, and co-occurrence. Using this data, we were able to determine which locations were most active in researching machine learning applications for concrete, where the most influential authors were in terms of both output and citations and which articles garnered the most citations overall.

**Conclusion:**

ML has become a viable prediction method for a wide variety of structural industrial applications, and hence it may serve as a potential successor for routinely used empirical model in the design of concrete structures. The non-ML structural engineering community may use this overview of ML methods, fundamental principles, access codes, ML libraries, and gathered datasets to construct their own ML models for useful uses. Structural engineering practitioners and researchers may benefit from this article’s incorporation of concrete ML studies as well as structural engineering datasets. The construction industry stands to benefit from the use of machine learning in terms of cost savings, time savings, and labor intensity. The statistical and graphical representation of contributing authors and participants in this work might facilitate future collaborations and the sharing of novel ideas and approaches among researchers and industry professionals. The limitation of this systematic review is that it is only PubMed based which means it includes studies included in the PubMed database.

## Introduction

Innovation and carbon emissions have forced building firms to utilize an increasing amount of high-performance manufactured materials. High building materials provide better strength, ductility, durability, resistance to external forces, more ecologically friendly development, and cheaper costs in long term than typical construction products (Aiyer et al., 2014). High-performance construction materials may come with higher initial costs, their potential for long-term cost savings through improved performance, energy efficiency, and reduced maintenance can make them economically viable choices. It is possible for them to dramatically extend the useful life of construction structures and minimize the amount of time and money needed to maintain such buildings. Construction materials that are known for their high level of performance include high-strength polymeric materials, lightweight steel, and concrete nanocomposite reinforced with glass fibers. Concrete, a major building product, is one of the greatest user of virgin resources including sand, gravel, crushed stone, and fresh water and it consumes around 1.6 billion metric tons of Portland and altered Portland cement each year (Pathak & Siddique, 2012). The primary component of concrete, Portland cement, is an energy and resource hog. About 7% of the world’s total CO_2_ emissions come from the manufacture of cement, making it one of the two greatest sources of greenhouse gas. Research is underway to develop unique materials that improve the qualities of high-strength concrete in order to produce concrete high-performance and ecologically friendly (Pathak & Siddique, 2012; Uysal & Sumer, 2011).

Fly ash (FA) is becoming a popular alternative to Portland cement in concrete because it saves resources, lasts longer, costs less, and is good for the environment (Bingöl & Tohumcu, 2013). In addition to being good for the environment, fly ash improves the stability of both high strength concrete by making it easier to work with, making it stronger over time, making it more resistant to sulfate attacks and alkali-silica reactions, lowering the heat of hydration (Güneyisi, Gesoğlu & Özbay, 2010), making it less likely to shrink, making it last the same amount of time when it freezes and thaws, making it less porous, and making it less permeable (Güneyisi, Gesoğlu & Özbay, 2010; Gesoğlu, Güneyisi & Özbay, 2009). But the amount and type of fly ash used in concrete has to be planned and described correctly because fly ash is not made in a special way and cannot be controlled by strict rules. At the end of the 1940s, FA was sold on the national market of concrete. It was known that using FA in concrete would improve the performance of high-volume FA (HVFA) concrete by making it easier to work with (thanks to the ball-bearing effect of spherical particles), making it stronger over time, cheaper, and more durable.  Since FA is a waste product, it cuts down the total cost of making concrete by a large amount (Sukumar, Nagamani & Srinivasa Raghavan, 2008; Bouzoubaâ & Lachemi, 2001; Siddique, 2011). FA will have different qualities from plant to plant since it is not made in a specific way and FA must confirm to certain standards like any other ingredient for concrete. In other words, its properties are dependent on the characteristics of pulverized coal and how the pulverization process is done in power plants that make electricity. Over time, HVFA concrete may get close to the strength of Portland cement concrete (PCC). FA reduces the HVFA cementitious materials’ internal curing thermostat, drying shrinkage, and porous air vacuum. This shows HVFA concrete compositions are may be as durable as or greater than PCC (Jiang & Malhotra, 2000). FA, due to its spherical shape and flat texture of granules, its particulate wrapping effect, and the safeguarding of cement particles from flocculation through opposite charges, can lead to increased deformation and durability related to porosity. These factors collectively contribute to making FA an essential component in concrete, as supported by references (Topçu & Sarıdemir, 2008; Kurda et al., 2018).

An artificial intelligence (AI) subfield known as machine learning (ML) focuses on teaching computers the skill of making predictions using existing datasets and methods. The most essential benefit is that computers may learn and develop automatically rather than being supervised learning (Han, Kamber & Pei, 2013). It was not until the 1990s that ML became the most prospering branch of AI, and began to grow, despite its 1943 birth and 1959 coinage. Since it is crucial in numerous applications of the real world, including voice and picture recognition, medical diagnosis, traffic warnings, and self-driving vehicles, ML has also become one of our generation’s most popular buzzwords in the technological industry. ML according to the learning experience, supervised, unsupervised, and reinforcement learning are all examples of AI (Witten, Frank & Hall, 2016). The most fundamental kind of ML is supervised learning, in which a labeled data set is used for an algorithm in teaching. Structural engineering is a branch of engineering that deals with the design and study of structures that are capable of supporting loads. In structural engineering, this technique has been extensively utilized for damage identification (classification issues) and strength forecasts (regression problems). Unsupervised learning, on the other hand, uses an algorithm that is trained on an unlabeled collection of data. As a result of this, the algorithm is honed using the reinforcement learning approach. More and more machine learning techniques are being used in structural engineering. These include neural networks (NN), decision trees (DT) and boosting algorithms (BA), regression analysis (RA), and support vector machines (SVM) (Witten, Frank & Hall, 2016; Parsons, 2010; Karbhari & Lee, 2009). Engineering design has utilized meta-models (sometimes called surrogate models) to speed up the calculation of black-box ML models with a relaxed level of accuracy in an effort to save computational time. It is open an interpretation model that is trained to mimic the forecasts of a black-box ML model. That is why they are called “surrogates”: basic analytical models that act like complicated machine-learning models (Parsons, 2010). A time-consuming calibration procedure is required for structural analysis and design approaches when dealing with severe actions that display extremely nonlinear behavior. These methods are also difficult to execute in practice. To reduce time and effort, ML might be a viable option (Sukumar, Nagamani & Srinivasa Raghavan, 2008; Topçu & Sarıdemir, 2008; Karbhari & Lee, 2009). In 1991, Adeli and Hung used an artificial neural network (ANN) to construct steel beams in one of the earliest ML applications in structural engineering (Hung & Adeli, 1991). Structural engineering was in its infancy at the time because of the limits of ML methods and computational capacity. In the early stages of structural engineering applications, this is shown by the fact that just a few relevant publications were published annually (Hung & Adeli, 1991; Adeli, 2001). It is also difficult to use machine learning in structural engineering since there are not enough test datasets for ML models. Structural analysis research has taken the required efforts to overcome this obstacle by developing databases to gather data from structural analysis testing. There are about 250 datasets from more than 50,000 trials housed in the DataCenterHub repository platform (Hajela & Berke, 1991; Catlin et al., 2018). Network for Earthquake Engineering Simulation (NEEShub) (Hacker, Eigenmann & Rathje, 2013) is a cyberinfrastructure system for earthquake engineering and catastrophe risk assessment. DesignSafe (Pinelli et al., 2020) is an extension of the NEEShub. NEEShub datasets for seismic design can be obtained from DataCenterHub (DEEDShub, 2017), as well as image databases for crack damage detection (*e.g.*, Structural ImageNet with more than 10,000 images, PEER Hub ImageNet) (Gao & Mosalam , 2020) established by the Pacific earthquake engineering research (PEER) center with more than 36,000 images, bridge crack library with more than 11,000 images, *etc*.). Advances in ML methods have also been made in the field of structural engineering (Rathje et al., 2017). For big datasets, BA approaches like extreme gradient boosting (XGBoost) (Chen & Guestrin, 2016) and classified gradient boosting (CatBoost) are particularly powerful tools. CNN is considered state-of-the-art ML technology because of its speed in identifying structural fracture damage. AutoML-Zero, a novel ML approach developed by the Google team recently, can progress autonomously without human involvement. TensorFlow and Keras from Google and PyTorch from Facebook are two examples of open-source ML libraries that provide hands-on ML algorithms and ready-to-run tools for construction applications (Real et al., 2020; Sun, Burton & Huang, 2021).

The scientific world has seen a significant raise in the application of ML in engineering structures, notably over the duration of last five years, with an evident exponential surge in the number of articles in both journals and conferenceseach year rapid evolution of ML algorithms and processing capacity. However, the use of ML in construction applications is currently relatively restricted. The industry has created ML-powered tools to produce alternative designs that fulfill the criteria of end-users as one of the real-world uses of creative models. Many recent review publications have addressed this topic, but they only focused on a specific area of engineering structures (*e.g.*, systemic implementation and quality, building system for fire; tangible property; cement mix proportions; capacity forecasting of concrete buildings; and layout and safety checks of bridges) only but instead structural engineering needs a complete assessment of all aspects (Fan et al., 2021; Mirrashid & Naderpour, 2021; Salehi & Burgueño, 2018; Mishra, 2021). The aim of this systematic review is to summarize maximumstudies in recent years implementing the approach of machine learning on the prediction in structural engineering but in consideration of the limitation applied to concrete as material because this is extensively used material in the construction industry (Pathak & Siddique, 2012).

### Rationale

The rationale for conducting this systematic review on machine learning applications in concrete is driven by the need to address the challenges and limitations of traditional structural analysis and design approaches. The construction industry heavily relies on concrete, which consumes significant amounts of virgin resources and plays a crucial role in building infrastructure. However, the conventional methods used for structural analysis often require time-consuming calibration procedures and struggle to handle severe actions with highly nonlinear behavior. Therefore, there is a need to explore alternative approaches that can reduce time and effort while improving accuracy and efficiency. Machine learning has shown promise in various fields, and its potential application in concrete structural engineering warrants investigation to identify its benefits and limitations.

The intended audience for this systematic review includes both structural engineering practitioners and researchers in the field of concrete construction. Structural engineers who are interested in exploring new approaches for structural analysis and design will find value in the overview of machine learning methods, principles, and available resources provided in this article. Researchers in the field of concrete and machine learning will benefit from the summary of existing studies, knowledge mapping, and identification of influential authors and nations. Additionally, professionals in the construction industry, including contractors, developers, and project managers, can gain insights into the potential benefits of machine learning in terms of cost savings, time efficiency, and labor intensity. Overall, this review aims to bridge the gap between traditional structural engineering practices and the emerging field of machine learning, providing a valuable resource for those seeking to incorporate ML methods into concrete applications.

### Problem statement and research question

This is the most recent and state-of-the-art review on the application of machine learning techniques to predict the properties of different types of concrete. The goal is to conduct a literature review to summarize all the work done on the prediction of all the mechanical properties of concrete. This literature review will help future researchers to opt for the best algorithm for their concrete and later compare them with the work already done in this area.

## Methodology for Conducting Systematic Review

Recent decades have witnessed the production of civic studies in huge numbers. As a result of this heterogeneity, the research provided might affect the investigation in a variety of ways, which complicates evidence and makes it more difficult to draw conclusions (Bello et al., 2015). Systematic review and meta-analysis (SR/MAs) is the evidence-based pyramid’s highest level of proof. To keep doctors and nurses up to date on the latest evidence-based medicine, it is possible to use an organized, well-managed SR/MA. As a result of our research, we discovered that the most important processes in a systematic review remain framing, discovering relevant studies *via* requirements construction and article search, assessing the quality of the studies utilized, summarizing data, and interpreting conclusions. The majority of issues may be solved by a researcher without any prior knowledge of the subject matter (Tawfik et al., 2019). For this study, we followed the Preferred Reporting Items for Systematic Reviews & Meta-Analysts (PRISMA) criteria (Liberati et al., 2009).

### Search engine and keywords

**Figure 1:**
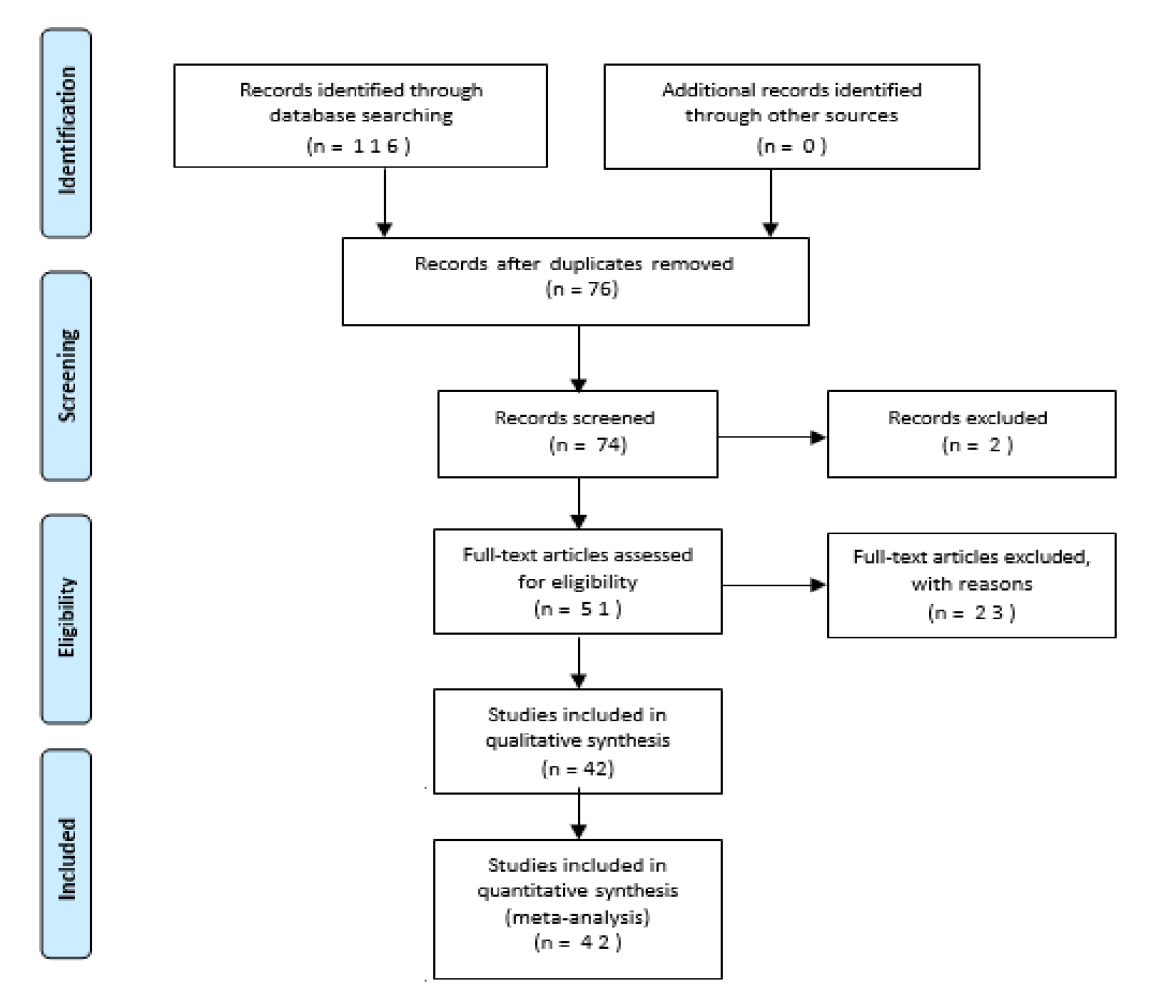
PRISMA-based flowchart showing the studies recruitment process.

First, a set of keywords has been formulated which is given below to search the PubMed database for the relevant studies, then after removing duplicates and the inclusion and exclusion criteria discussed in Table 1 were applied to the rest of the studies which then resulted in narrowing the studies from 116 to 42 (Fig. 1). Then for the deeper search and in order to get the most possible and accurate results, the following keywords were also divided into different sets.

**Table 1:** Inclusion and exclusion criteria for the recruitment of studies are discussed in detail.

**Inclusive criteria**	**Exclusive criteria**
• Concrete was used as the primary material in the study. • Studies that use any machine learning algorithm to predict the properties. • Studies that are published are either original articles or review articles in any conference proceeding or journal.	• The material used in some of the studies was not concrete. • Studies that use any other method other than machine learning for the prediction. • Studies that are not published are either original articles or review articles in any conference proceeding or journal.

 • (concrete technology) AND (mechanical OR durability OR compressive strength OR flexural strength OR modulus of elasticity OR tensile strength) AND (“computer vision” OR “neural network” OR “artificial intelligence” OR “pattern recognition” OR “machine learning”).

### Eligibility criteria

Table 1 outlines inclusion and exclusion criteria for a study, likely related to the prediction of concrete properties using machine learning algorithms. These criteria are used to define the scope of the study and to determine which studies should be included in the analysis and which should be excluded. The inclusive criteria define the characteristics that studies must have to be considered for analysis (focus on concrete, use of machine learning, and publication in conferences or journals). The exclusive criteria define the characteristics that would lead to the exclusion of studies from the analysis (focus on non-concrete materials, use of methods other than machine learning, and lack of appropriate publication types). These criteria help ensure that the study’s scope remains relevant and focused on the specific research objectives.

### Flowchart

## Results

It was aimed to ensure a rigorous and focused selection process to identify the most relevant studies for our analysis. Starting with an initial pool of 116 articles, the authors employed a systematic approach to narrow down the selection to the final set of 42 articles that were included in our study. In addition to the criteria listed in the table, which encompassed aspects such as the use of concrete as the primary material, the application of machine learning algorithms, and publication in recognized conferences or journals, we also considered several other specific conditions to refine the selection.

Firstly, the authors assessed the alignment of the studies with our research objectives. Carefully examined the research questions, objectives, and methodologies presented in each article to ensure that they were directly relevant to our investigation of predicting concrete properties using machine learning techniques. Secondly, the authors scrutinized the quality and reliability of the machine learning methods employed in the studies. We favored articles that demonstrated a clear understanding of machine learning principles, appropriate use of algorithms, and thorough validation of their predictive models. Lastly, the authors considered the diversity of the approaches and datasets used across the articles. It was aimed to capture a comprehensive spectrum of machine learning techniques and concrete property predictions, ensuring a well-rounded representation of the field. The final selection of 42 articles emerged as a robust and comprehensive collection that provided a strong foundation for our analysis. This stringent selection process bolstered the reliability and validity of our findings and conclusions.

Table 2 provides a list of research studies on the application of machine learning algorithms in predicting the properties of different types of concrete. The table includes the authors, year of publication, type of concrete, property predicted, number of input parameters, machine learning algorithms used, and reported outcomes. Some of the machine learning algorithms used in these studies include boosted decision tree regression, support vector machine, artificial neural network, genetic algorithm-optimized backpropagation neural network, multi-expression programming, linear regression, and extreme gradient boosting. The properties predicted include compressive strength, split tensile strength, modulus of elasticity, and static modulus.The reported outcomes include correlation coefficients, root mean square error, mean absolute error, accuracy, coefficient of determination, mean absolute percentage error, and mean squared error. The studies vary in the number of input parameters, ranging from 1 to 10. Some studies used conventional artificial neural networks, adaptive neuro-fuzzy inference, and tabular generative adversarial networks to predict the properties of concrete.

**Table 2:** Summarized details of the studies recruited after conducting PRISMA-based systematic review.

**Reference**	**Author**	**Year**	**Material**	**Properties**	**Input parameter**	**Machine learning algorithm**	**Reported outcomes**
Latif (2021)	Latif et al.	2021	Environmentally friendly concrete	Compressive strength	8	-Boosted decision tree regression (BDTR) -Support vector machine (SVM)	*R* = 0.86 RMSE = 6.19 MAE = 4.91 RSR = 0.37
Iqbal et al. (2021)	Iqbal et al.	2021	Concrete waste foundry sand (CWFS).	-Split tensile strength (ST) -Modulus of elasticity (E)	4	Multi-Expression Programming (MEP)	ST: *R* = 0.93 RMSE = 0.36 MAE = 0.28 RSE = 0.21 Accuracy = 0.051E *R* = 0.96 RMSE = 2.13 MAE = 1.70 RSE = 0.17 Accuracy = 0.032
Du et al. (2021)	Du et al.	2021	High-performance self-compacting concrete	-Compressive strength	?	Genetic algorithm (GA)-optimized backpropagation neural network (BPNN) model	BPNN: Correlation coefficient = 0.967 RMSE = 3.703 GA-BPNN: Correlation coefficient = 0.979 RMSE = 2.972
Safiuddin et al. (2016)	Saifuddin et al.	2016	Journal	Concrete	?	Artificial neural networks (ANN)	Coefficient of determination (R^2^) = 0.9486
Hadzima-Nyarko et al. (2019)	Hadzima-Nyarko et al.	2019	Waste Rubber Concrete	-Compressive strength	6	Artificial neural networks (ANN)	highest R value of 0.96 and 0.98 for the train and test data, respectively, an achieved the lowest RMSE and MAPE values (4.8 and 20.2 for the train data, respectively, and 3.78 and 21.6 for the test data
Van Dao et al. (2019)	Dao et al.	2019	Geopolymer Concrete	-Compressive strength	4	-Adaptive neuro fuzzy inference (ANFIS) -Artificial neural network (ANN)	-ANFIS (MAE = 1.655 MPa, RMSE = 2.265 MPa, and R2 = 0.879) -ANN (MAE = 1.989 MPa, RMSE = 2.423 MPa, and R2 = 0.851)
Ziolkowski & Niedostatkiewicz (2019)	Ziolkowski et al.	2019	Concrete	-Compressive strength	?	-Artificial neural network (ANN)	?
Yoon et al. (2019)	Yoon et al.	2019	Lightweight Aggregate Concrete	-Compressive strength -Elastic modulus	10	-Artificial neural network (ANN)	CS: MAE% = 14.5% Correlation coefficient = 0.930 E: MAE% = 8.5% Correlation coefficient = = 0.977
Abambres & Lantsoght (2019)	Abambres et al.	2019	Concrete	-Compressive strength	1	-Artificial neural network (ANN)	AVG = average = 1.00 STD = standard deviation = 0.02 COV = co-efficient of variation = 1.69%
Van Dao et al. (2020)	Dao et al.	2020	Foamed Concrete	-Compressive strength	3	-Conventional Artificial Neural Network (C-ANN)	R^2^ = 0.972 RMSE = 0.140 MAE = 0.114
Park et al. (2020)	Park et al.	2020	Concrete	-Static modulus –Compressive strength	6	-SVM -Ensemble -ANN -Linear regression	SVM: MSE = 12.75 MAPE = 13.71 Ensemble: MSE = 11.54 MAPE = 14.31 ANN: MSE = 29.50 MAPE = 15.47 LR: MSE = 44.77 MAPE = 29.59
Marani, Jamali & Nehdi (2020)	Marani et al.	2020	Ultra-high-performance concrete (UHPC)	–Compressive Strength	8	-Tabular Generative Adversarial Networks (TGAN) -Tree-Based Ensembles	TGAN: MAE = 5.46 RMSE = 8.47 *R*^2^ = 0.95 Ensemble: MAE = 6.72 RMSE = 8.41 *R*^2^ = 0.95
Wan, Xu & Šavija (2021)	Wan et al.	2021	Concrete	-Compressive Strength	-8 original features -6 Principal Component Analysis (PCA) Features -6 Manual features.	-Linear regression (LR) -Support Vector Regression (SVR) -Extreme Gradient Boosting (XGBoost) - Artificial Neural Network (ANN),	LR: MSE = 44.90 *R*^2^ = 0.84 SVR: MSE = 25.8 *R*^2^ = 0.91 XGBoost: MSE = 33.87 *R*^2^ = 0.87 ANN: MSE = 26.4 *R*^2^ = 0.91
Ahmad et al. (2021b)	Ahmad et al.	2021	Fly Ash Based Concrete	–Compressive Strength	8	-Decision tree (DT) -Ensemble approach -Gene Expression Programming (GEP)	DT: MAE = 3.89 MSE = 36.01 RMSE = 6.00 DT-bagging: MAE = 3.113 MSE = 16.28 RMSE = 4.03 GEP: MAE = 3.47 MSE = 29.91 RMSE = 5.46
Ali Khan et al. (2021)	Khan et al.	2021	Geopolymer Concrete	–Compressive Strength	9	-Gene Expression Programming (GEP)	RMSE = 2.64 MAE = 2.057 RSE = 0.06 *R* = 0.9643
Huseien et al. (2021)	Huseien et al.	2021	Self-healing concrete	Mechanical and durability properties	8	Artificial Neural Network (ANN)	MSE = 3.72 ME = 0.89 MAE = 1.11 RMSE = 1.93
Mhaya et al. (2021)	Mhaya et al.	2021	Waste rubber tire crumbs (WRTCs)-based concrete	–Compressive Strength	6	Artificial Neural Network (ANN)	MSE = 189.69 ME = 3.052 MAE = 8.139 RMSE = 13.773
Ahmad et al. (2021d)	Ahmad et al.	2021	Concrete	–Compressive Strength	10	-AdaBoost -Random forest (RF) -Decision tree (DT)	AdaBoost: *R*^2^ = 0.938 RSR = 0.248 MAPE = 12.52 RRMSE = 11.62 RF: *R*^2^ = 0.935 RSR = 0.256 MAPE = 13.076 RRMSE = 11.661 DT: *R*^2^ = 0.911 RSR = 0.324 MAPE = 16.100 RRMSE = 14.753
Ahmad et al. (2021c)	Ahmad et al.	2021	Concrete	–Compressive Strength	?	-Decision tree (DT) -Artificial neural network (ANN) -Bagging -Gradient boosting (GB)	DT: MAE = 7.54 MSE = 112.3 RMSE = 10.79 Bagging: MAE = 5.65 MSE = 61.08 RMSE = 7.81 GB: MAE = 6.93 MSE = 85.1 RMSE = 9.24 DT: MAE = 9.15 MSE = 121.66 RMSE = 11.03
Kovačević et al. (2021)	Kovačević et sl.	2021	Self-Compacting Rubberized Concrete	–Compressive Strength	11	-Multilayered perceptron artificial neural network (MLP-ANN) -Ensembles of MLPANNs,	MLPANN: RMSE = 7.44 MAE = 5.54 *R* = 0.8481 Ensemble MLPANN: RMSE = 3.68 MAE = 2.80 *R* = 0.9615
Song et al. (2021)	Song et al.	2021	Ceramic Waste-Based Concrete	–Compressive Strength	5	-Decision tree (DT) -Artificial neural network (ANN)	DT: MAE = 6.94 MSE = 20.76 RMSE = 4.55 ANN: MAE = 6.12 MSE = 17.98 RMSE = 4.29
Farooq et al. (2021)	Farooq et al.	2021	Self-Compacting Concrete Modified with Fly Ash	–Compressive Strength	7	-Artificial neural network (ANN) -Support vector machine (SVM) -Gene Expression Programming (GEP)	ANN: *R* = 0.95 RMSE = 4.56 MAE = 3.81 SVM: *R* = 0.93 RMSE = 4.49 MAE = 3.29 GEP: *R* = 0.93 RMSE = 4.8 MAE = 3.92
Ahmad et al. (2021e)	Ahmad et al.	2021	Concrete Containing Supplementary Cementitious Materials	–Compressive Strength	8	-Bagging -AdaBoost -Gene Expression Programming (GEP) -decision tree (DT)	Bagging: MAE = 3.257 MSE = 20.566 RMSE = 4.53 AdaBoost: MAE = 5.12 MSE = 47.37 RMSE = 6.88 GEP: MAE = 5.24 MSE = 50.69 RMSE = 7.12 DT: MAE = 5.88 MSE = 57.30 RMSE = 7.57
Tosee et al. (2021)	Tosee et al.	2021	Environmentally Friendly Concrete Modified with Eggshell	–Compressive Strength	4	Hybrid ANN-SFL (artificial neural network-Shuffled Frog Leaping)	MSE = 0.42 AAE = 0.040 VAF = 94
Xu et al. (2021)	Xu et al.	2921	-Concrete	–Compressive Strength	7	-Support vector regression (SVR) -AdaBoost -random forest	SVR: MAE = 3.329 RMSE = 5.325 AdaBoost: MAE = 2.94 RMSE = 3.90 RT: MAE = 2.223 RMSE = 3.183
Isleem et al. (2021)	Isleem et al.	2021	GFRP-Reinforced Concrete	-Axial load-axial strain -Confinement of columns -Ductility -Hardening behavior	6	–Artificial neural network (ANN) - Finite Element (FEM)	?
Nafees et al. (2021)	Nafees et al.	2021	Silica Fume-Based Green Concrete	Split Tensile Strength; compressive strength	5	- Multilayer perceptron neural networks (MLPNN) -Adaptive neural fuzzy detection systems (ANFIS) -Genetic expression programming (GEP)	MLPNN: 0.85; 0.90 ANFIS: 0.91; 0.92 GEP: 0.97; 0.93
Khokhar et al. (2021)	Khokhar et al.	2021	Fiber Reinforced Concrete	-Compressive Strength -Tensile Strength -Strain- Hardening -Tensile Strain Capacity	15	- Artificial Neural Networks (ANN) -Support Vector Machine (SVM) -XGBoost	ANN: Accuracy = 96.3% SVM: Accuracy = 94% XGBoost: Accuracy = 98.4%
Imran et al. (2022)	Imran	2022	Eco-Friendly Concrete	–Compressive Strength	6	-Multivariate polynomial regression (MPR) -Linear regression (LR) -Support vector machine (SVM)	MPR: *R*^2^ = 0.818 RMSE = 4.6 LR: *R*^2^ = 0.676 RMSE = 6.053 SVM: *R*^2^ = 0.495 RMSE = 7.38
Almohammed et al. (2022)	Almohammed et al.	2022	Bacterial concrete	–Compressive Strength	8	-Multiple Linear Regression (MLR) -Random Forest (RF) -Support vector Regression (SVR) -M5P Model -Random Tree	MLR: *R*^2^ = 0.88 RMSE = 4.87 MAE = 3.96 RF: *R*^2^ = 0.97 RMSE = 2.29 MAE = 1.81 SVR: *R*^2^ = 0.98 RMSE = 1.94 MAE = 1.52 RT: *R*^2^ = 0.96 RMSE = 2.82 MAE = 2.49 M5P: *R*^2^ = 0.94 RMSE = 4.88 MAE = 2.88
Shang et al. (2022)	Shang et al.	2022	Recycled coarse aggregate based concrete	Splitting tensile strength; Compressive Strength	9	-Decision tree (DT) -AdaBoost	DT: MAE = 3.58; 0.31 MSE = 11.02; 0.29 RMSE = 3.32; 0.54 AdaBoost: MAE = 2.33; 0.30 MSE = 7.8; 0.20 RMSE = 2.79; 0.45
Candelaria, Kee & Lee (2022)	Candelaria et al.	2022	Concrete	–Compressive Strength	8	-Artificial neural network (ANN) -Support vector machine (SVM) -Gaussian process regression (GPR) - Multi-Variate Regression	ANN: *R*^2^ = 0.97 RMSE = 9.4 MAE = 9.414 SVM: *R*^2^ = 0.95 RMSE = 18.04 MAE = 12.33 GPR: *R*^2^ = 0.94 RMSE = 18.14 MAE = 13.072 MVR: *R*^2^ = 0.93 RMSE = 9.5 MAE = 17.215
Ahmed et al. (2022)	Ahmed et al.	2022	Geopolymer concrete	-Compressive Strength	14	-Linear regression (LR) -Multinominal logistic regression (MLR) -Nonlinear regression (NLR)	*R*^2^ = 0.853 RMSE = 6.82
Najm et al. (2022)	Najm et al.	2022	Waste ceramic concrete (WOC)	Tensile strength; compressive strength	11	-Artificial neural networks (ANN)	*R*^2^ = 0.9988; 0.9687 MSE = 0.22; 1.8899 RMSE = 0.4699; 1.3744 MAE = 0.469; 1.2279
Hung & Adeli (1991)	Hung & Adeli	1991	Recycled aggregate concrete (RAC)	Compressive strength; Flexural strength	12	-Gradient boosting -Random forest (RF)	GB: MAE = 4.77; 0.642 RMSE = 6.9; 1.199 RF: MAE = 4.19; 0.560 RMSE = 5.6; 0.85
Ray et al. (2022)	Ray et al.	2022	Concrete made (stone dust and nylon fiber)	Strength	8	-Artificial neural networks (ANN)	*R* = 0.95 *R*^2^ = 0.90 MSE = 0.09 MAE = 0.20 AE = 0.04
Ilyas et al. (2021)	Ilyas et al.	2021	CFRP Confined Concrete	-Strength	8	-Multi Expression Programming (MEP)	RMSE = 7.71 RSE = 0.009 MAE = 6.33 RRMSE = 0.010 *R* = 0.9953
Gunasekara et al. (2021)	Gunasekara et al.	2021	High Calcium Fly Ash Geopolymer Concrete	-Compressive strength	5	-Artificial neural networks (ANN)	?
Ahmad et al. (2021a)	Ahmad et al.	2021	Geopolymer concrete (GPC)	-Compressive strength	9	-Artificial neural networks (ANN) -Boosting algorithm -Ada boost	ANN: MAE = 3.86 MSE = 20.16 RMSE = 4.49 Boosting algorithm: MAE = 1.69 MSE = 4.16 RMSE = 2.04 AdaBoost: MAE = 2.16 MSE = 6.84 RMSE = 2.62
Amin et al. (2022)	Amin et al.	2022	Fiber-reinforced polymer (FRP) reinforced Concrete	-Flexural Strength	9	-Decision tree (DT) -Gradient boosting tree (GBT)	DT: *R* = 0.92 MAE = 10.32 RMSE = 19.92 GBT: *R* = 0.94 MAE = 11.25 RMSE = 16.36
Khalaf, Kopecskó & Merta (2022)	Khalaf at al.	2022	Fly Ash Geopolymer Concrete	-Compressive strength	11	Optimized Neural Network Model	MSE = 166.0 R% = 97.5
Nafees et al. (2022)	Nafees et al.	2022	Plastic Concrete	-Compressive strength	9	Ensemble boosting	*R* = 0.814

**Notes.**

? not reported.

## Discussion and Limitations

**Figure 2:**
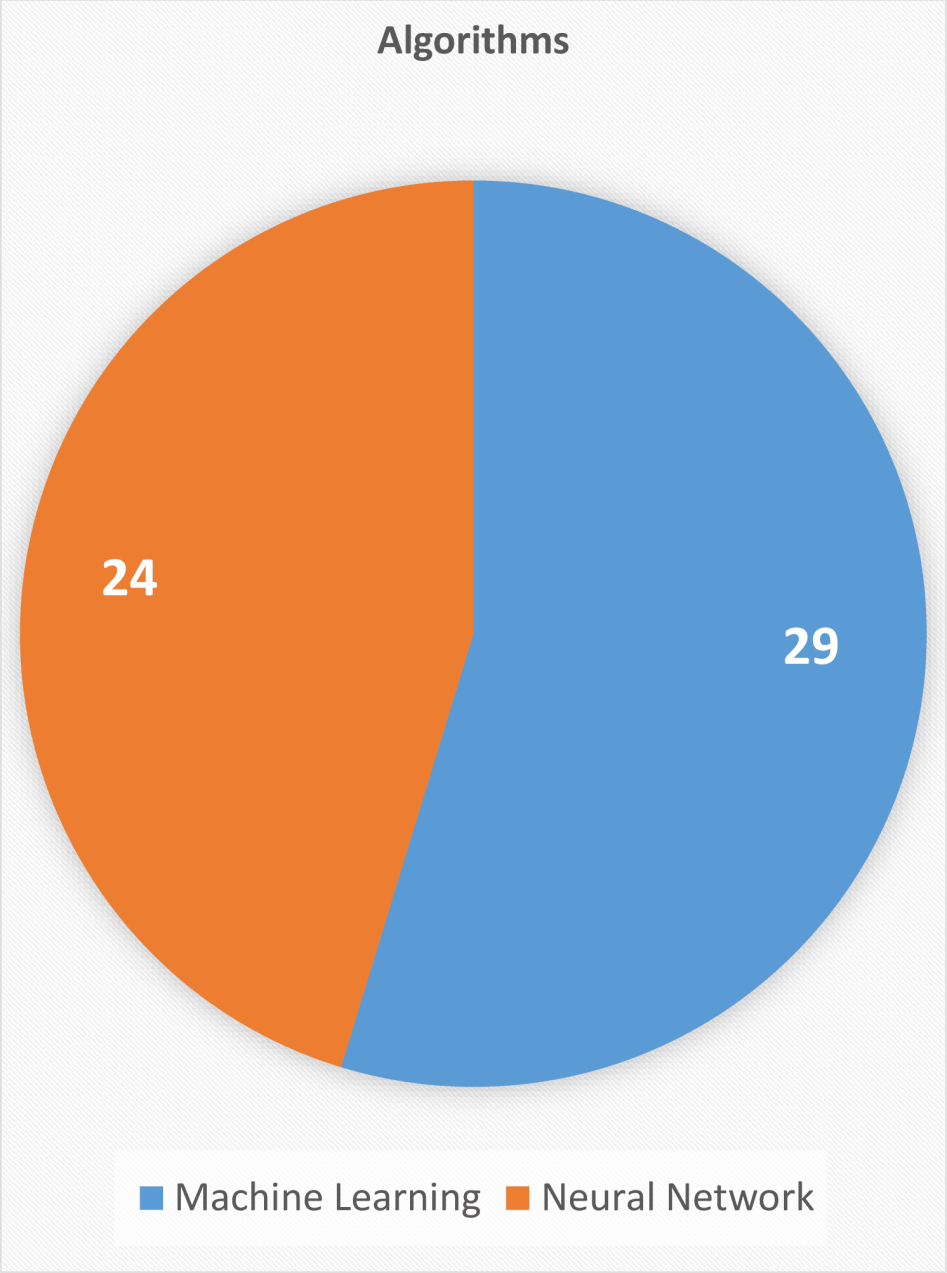
Pie chart of studies showing number of ML and NN techniques used in the selected studies.

**Figure 3:**
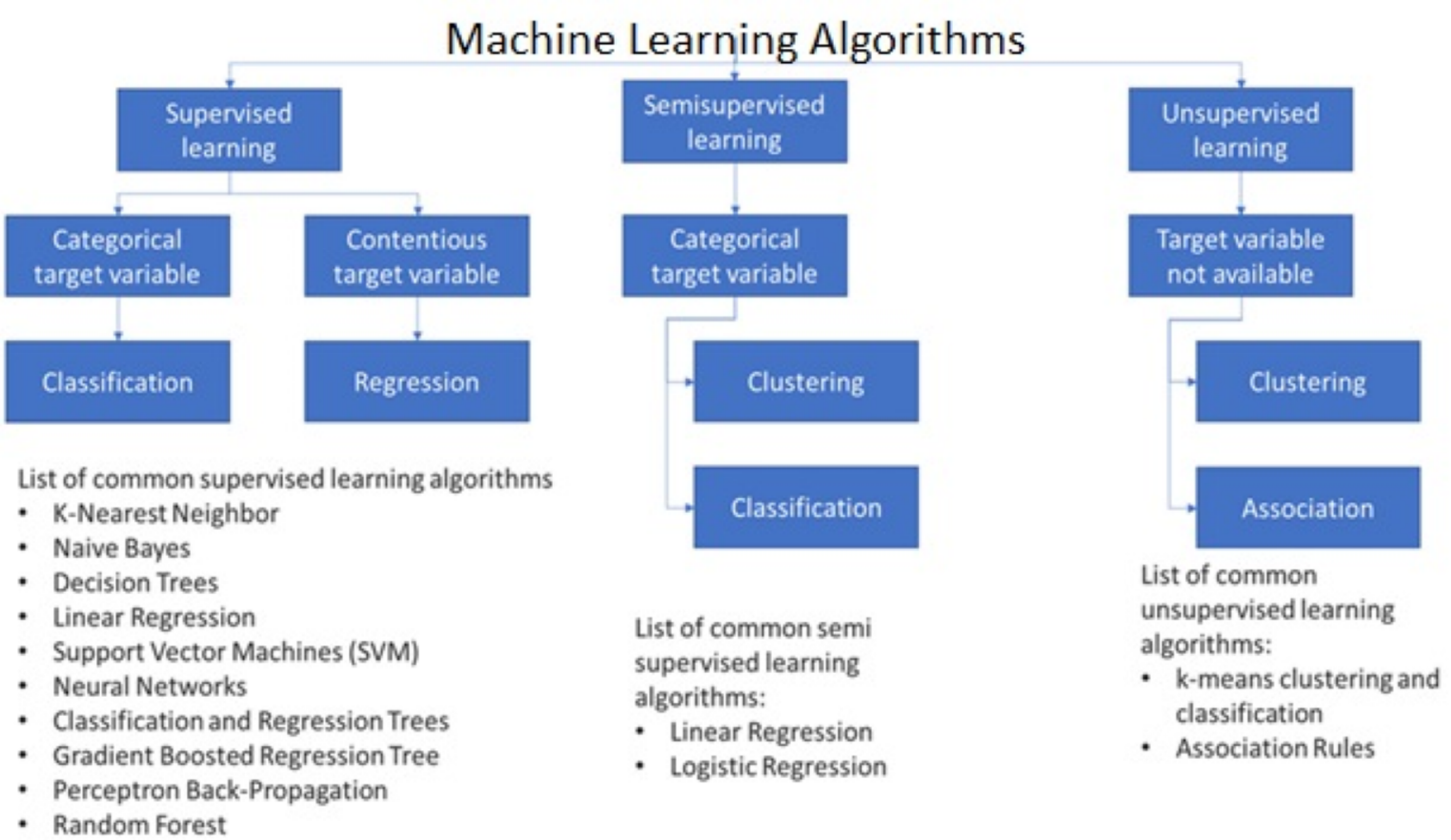
Classification of machine learning algorithms on the basis of their learning types (Aldahiri, Alrashed & Hussain, 2021).

In Fig. 2, we can see that 55% of the authors prefer applying supervised machine learning methods while 45% of the authors opted deep learning neural networks. But it is difficult to say which one is better although the highest accuracy achieved was through artificial neural network (Arciszewski, Mustafa & Ziarko, 1987). Three decades ago, the initial application of machine learning techniques was to try out several existing approaches to simple tasks. After then, more complicated issues began to be considered. Monitoring structural health, evaluating concrete qualities, and formulating new mixes are some of the most prevalent uses (Stone, Blockley & Pilsworth, 1989; Reich, 1997). In this part, we will take a look at how machine learning (Fig. 3) approaches have been implemented in these two scenarios.

### Structural health monitoring

Civil constructions are subject to structural degradation as a result of their usage and environment. For the assurance of assure public safety and the in-service construction dependability, the Structural Health Monitoring (SHM) system is essential for early detection of structural problems. Dynamic response assessments separated at periodic intervals are used to monitor a component over time, damage-sensitive characteristics are recovered, and then the derived features are statistically examined to determine the present health condition of the system (Khoa et al., 2014). Long-span bridges, massive dams, and towering buildings are among the structures where the SHM system has been widely deployed, allowing for a seamless transition from time-based to situation management. Model-driven or data-driven techniques have both been used in recent studies in this area of interest. As a result of this method, it is possible to detect structural deterioration by comparing measured data to data generated by a computer model of the structure (typically based on finite element analysis (FEA)). Due to the repetitive examination of a simulation software model, this technique is computationally intensive (Jin, Cho & Jung, 2015; Salazar et al., 2015; Cha & Buyukozturk, 2014; Ranković et al., 2012). It is also possible that in actuality, a measurement simulation may not be available at all times or accurately represent the real structure’s performances in every case. Because of this, FEA findings are typically insufficient to accurately measure structural health. A strategy based on data rather than models generates a model *via* the use of observed data and then compares the model’s responses to those measured in order to discover damage. This method employs machine learning techniques, such as pattern recognition. It is becoming more possible to install large and dense sensor networks for SHM because to recent advancements in sensing methods, and wireless communication. As a result, continuous and real-time damage identification is made much easier with the data-driven method (Aldahiri, Alrashed & Hussain, 2021). To identify structural damage, machine learning algorithms are often used in conjunction with supervised learning, which relies on examples of both healthy and damaged data. Structural damage detection may benefit from the resilience and efficiency of single machine learning method such as support vector machine, neural networks, and support vector regressions, as well as the genetic algorithms (GA). For various challenges in the SHM sector, hybrid approaches such as the multi-objective genetic algorithm (MOGA), neuro-fuzzy (NF), and wavelet neural network (WNN) have also been presented. All investigations proved the accuracy of machine learning-based models and their better performance over model-driven methods (Ranković et al., 2012; Taffese & Sistonen, 2017).

### Properties of mix design concrete

It can be seen in Table 2 that so many researchers contributed to predict the mechanical properties of the concrete mix with different substances like fly ash, foundry sand, or rubber waste using ML algorithms. Concrete buildings are designed with mechanical qualities including compressive strength, elastic modulus, splitting tensile strength, and shear strength in mind. Predicting the compressive strength of concrete by linear or non-linear regression equations saves both time and money (Chou et al., 2014). Elastic modulus measurement is difficult and time-consuming. Stress–strain relations of cementitious materials under compression are often used to get this information (Atici, 2011; Behnood, Verian & Modiri Gharehveran, 2015). The compressive strength of concrete is typically used to estimate the splitting tensile strength of concrete because of its complexity, expense, and time-consuming nature. Based on experimental data, regression models for shear strength of RC components are also applied. In the past, the mechanical characteristics of concrete were evaluated using a set equation that was based on a small amount of experimental data and variables. They are only useful for describing the results of their own experiments used to calibrate them. The model coefficients and the equation’s form must be updated if the original data is changed. To determine fresh concrete’s mechanical qualities, standard models may not be appropriate since the link between components and concrete characteristics is particularly nonlinear for certain concrete kinds. A widely agreed-upon mathematical model is also difficult to come by. A concrete structure’s long-term performance may be evaluated by looking at its dry shrinkage, another important feature of concrete. Several empirical equations for shrinkage estimation have been developed in various codes such as ACI and CEB throughout the last five decades. Dry shrinkage in concrete is affected by a variety of parameters, including its composition, the size of the specimen, and the quality of its ingredients. Using these calculations may be problematic in certain situations. Components and their relative proportions are determined in order to manufacture concrete that fulfills required strength, workability and durability at a low cost while yet delivering a high quality product. As an extension of previous practice, concrete mix percentage algorithms are typically available in the form of empirical formulae or tables. As a consequence of this uncertainty, typical methods for determining concrete mix proportions are a trial-and-error exercise, which results in higher expenses as well as more time (Nazari & Sanjayan, 2015). Modeling concrete characteristics and mix design accurately and reliably may save time and money by providing engineers with the information they need. To circumvent the limitations of standard empirical regression models, machine learning methods have been used to represent these features. Construction of accurate and effective models for predicting the characteristics and mix design of several kinds of concrete, including fiber-reinforced polymer (FRP) concrete have been done by using machine learning techniques. Many machine learning methods are used in these investigations, including neural networks, genetic programming, fuzzy logic, support vector machines, and fuzzy inference systems (FIS). Machine learning approaches have been shown to be a strong tool for evaluating tangible qualities, regardless of the complexity, incoherence, or incompleteness of the data used. They are also a superior alternative for deciding on the right quantities of materials in concrete mixtures to achieve the appropriate strength and rheology (Chou et al., 2014; Atici, 2011; Behnood, Verian & Modiri Gharehveran, 2015; Dantas, Batista Leite & de Jesus Nagahama, 2013; Nazari & Sanjayan, 2015; Mermerdaş & Arbili, 2015; Tang et al., 2015). Reducing trial mixes results in an ecological and cost-effective mix design method.

### Artificial neural network

Parallel processing occurs in the brain’s neural network, which is a web of linked neurons that sends signals back and forth to process information. ANNs are a cutting-edge analytical technique that mimics the way the human brain thinks. Similar to other DoE approaches that take in numerous factors to forecast the response variable, ANNs may be employed mathematically to analyze multiple inputs and generate an output (Sharma, Rai & Dev, 2012). The input, hidden layer, and output layer are all parts of the ANN’s mechanism. It is here where data is entered. The output layer processes the data and provides the result *via* a system of connection weights. The inputs are fed into the process, and the process concludes with the output. A technique known as backward propagation is used to reduce the overall weight of the network’s connections. The discrepancy between the anticipated value and the actual value is believed to alter and change the mechanism of the hidden layer. It is important to understand the benefits and downsides of ANNs (Behnood & Golafshani, 2018). Due to its processing, errors may be tolerated, and complicated non-linear relationships between variables can be solved with ease using data analysis. ANNs have a distinct edge over pre-programmed computational models since they are able to learn from their own mistakes. It is also possible to overfit the data supplied by ANNs because of the intricacy of their solution (Sharma, Rai & Dev, 2012; Mohammed, Khed & Nuruddin, 2018).

Concrete compressive strength may be predicted using ANNs, which have a greater number of variables than previous DoE approaches. Analyzing many concrete experiments that all employ the same looking to upgrade is a unique use of ANNs thanks to their enhanced processing capability. Gupta (2013) who collected 32 data points from ten different publications on nano-silica-containing concrete, came up with an exact model for 28-day concrete compressive strength without having to do any experiments. Additionally, Asteris & Mokos (2020) utilized non-destructive test results from a thesis to train ANNs on 209 data sets to estimate concrete strength. Noorzaei, Hakim & Jaafar (2007) and Santosa & Purbo Santosa (2017) did a similar study utilizing the elements of concrete as variables and reached the same outcome. In terms of precision, regression analysis, particularly multiple non-linear regression, falls short in comparison to ANNs, as shown by the R2 value. When it comes to modeling self-compacting concrete, research found that the results of MLR outperformed those generated by ANNs. ANNs function best when given more data, and the low quantity of data in the study (*i.e.,* 15) may account for this. The R2 score alone should not be utilized to choose the optimal model. The root mean squared error (RMSE) of the ANNs model was much lower than the other models in another experiment on recycled aggregate concrete.

### Comparison and motivation of literature review

The PRISMA based methodology adaption for this systematic literature review has been taken from Zahid et al. (2022). This literature review is unique because it systematically summarizes the current state of research on the application of machine learning in the concrete industry, with a focus on structural analysis and design approaches. The review provides a comprehensive overview of the potential of ML to replace empirical models and reduce the time and effort required in the industry. It also provides an overview of ML methods, principles, access codes, libraries, and datasets that can be used by practitioners and researchers to develop their own ML models. Additionally, this review identifies the most active locations and influential authors in researching ML applications for concrete, which could facilitate future collaborations and sharing of novel ideas and approaches among academics. The statistical and graphical representation of contributing authors and nations can be useful for researchers and practitioners in identifying potential collaborators and networking opportunities. Overall, this review provides a valuable resource for researchers and practitioners in the concrete industry who are interested in exploring the potential of ML to improve their work. The systematic approach used in this review ensures that the information presented is comprehensive and unbiased, making it a valuable resource for anyone looking to learn more about the application of ML in the concrete industry.

## Conclusion

It can be concluded that the use of ML is being explored as a potential method to reduce the time and effort required for structural analysis and design approaches in the concrete industry. The abstract summarizes a systematic review of 42 studies that were conducted using a set of keywords and PRISMA guidelines. The review highlights the potential of ML to serve as a successor to the routinely used empirical models in the structural engineering community. The article also provides an overview of ML methods, fundamental principles, access codes, ML libraries, and gathered datasets that can be used by practitioners and researchers to construct their own ML models for useful applications. The construction industry can benefit from the use of ML in terms of cost savings, time savings, and labor intensity. The systematic review also identifies the most active locations and influential authors in researching ML applications for concrete, which could facilitate future collaborations and sharing of novel ideas and approaches among academics. However, the limitation of this review is that it only includes studies that are included in the PubMed database.

### Future trend

The great degree of accuracy in actual and predicted outcomes demonstrates the significance of these techniques in civil engineering. It is becoming increasingly common to use supervised ML techniques since they provide accurate outputs and reduce the amount of physical labor and overall project expense. In addition, it is vital to conduct laboratory experiments to compare the results of machine learning algorithms. In order to compare the results of different machine learning algorithms, it is also possible to alter or add input factors, such as the number of data points and the kind of material used, size of specimens, ambient conditions, curing settings, and data loading rate. For the sake of comparison, a variety of machine learning approaches may be used, including ANNs, SVMs, and boosting (Shang et al., 2022). Databases were used to calculate the compressive and split tensile strengths. As an alternative, additional input parameters and increasing the database may produce the required results. Silica Fume Concrete (SFC) is compressive and split tensile strength models have been created in this work. According to statistical characteristics, these models were able to accurately and reliably estimate SFC intensities. However, by using the same modeling parameters, MLPNN, ANFIS, and GEP models may be used to forecast concrete qualities including numerous different concrete ingredients. Based on input parameters, these models will be changed and the outcomes anticipated are largely dependent on the database used. The whale optimization algorithm, ant colony optimization, and particle swarm optimization are just a few examples of heuristic techniques that may be utilized in combination with machine learning to get optimum results. They may then be compared to this study’s methods. The upgraded and improved version of GEP is known as multi-expression programming (MEP). GEP’s limitations may be overcome *via* MEP analysis. To put it simply, MEP is given more attention when the complexity of the target expression is uncertain. There are exceptions, erroneous expressions, and even division by zero that can be handled by MEP. There are no infertile learners in the next generation since the gene is responsible for causing exceptions and then changing to an arbitrary terminal symbol. While MLPNN and ANFIS were used for the prediction of results, single learners were utilized in this study to anticipate results. Many different sub-models are built, and statistical parameters are used to pick the best one. This is known as an ensemble ML approach (Imran et al., 2022).

## Additional Information and Declarations

### Data Availability

The following information was supplied regarding data availability: This is a literature review.
